# Fucans, but Not Fucomannoglucuronans, Determine the Biological Activities of Sulfated Polysaccharides from *Laminaria saccharina* Brown Seaweed

**DOI:** 10.1371/journal.pone.0017283

**Published:** 2011-02-28

**Authors:** Diego O. Croci, Albana Cumashi, Natalia A. Ushakova, Marina E. Preobrazhenskaya, Antonio Piccoli, Licia Totani, Nadezhda E. Ustyuzhanina, Maria I. Bilan, Anatolii I. Usov, Alexey A. Grachev, Galina E. Morozevich, Albert E. Berman, Craig J. Sanderson, Maeve Kelly, Patrizia Di Gregorio, Cosmo Rossi, Nicola Tinari, Stefano Iacobelli, Gabriel A. Rabinovich, Nikolay E. Nifantiev, on behalf of the Consorzio Interuniversitario Nazionale per la Bio-Oncologia (CINBO), Italy

**Affiliations:** 1 Laboratorio de Inmunopatología, Instituto de Biología y Medicina Experimental (IBYME), Consejo Nacional de Investigaciones Científicas y Técnicas (CONICET) y Departamento de Química Biológica, Facultad de Ciencias Exactas y Naturales, Universidad de Buenos Aires, Ciudad de Buenos Aires, Argentina; 2 Department of Oncology and Neurosciences, University G. D'Annunzio Medical School and Foundation, Chieti, Italy; 3 V.N. Orekhovich Research Institute of Biomedical Chemistry, Russian Academy of Medical Sciences, Moscow, Russian Federation; 4 Consorzio Mario Negri Sud, Laboratory of Vascular Biology and Pharmacology, Santa Maria Imbaro, Chieti, Italy; 5 Laboratory of Glycoconjugate Chemistry, N.D. Zelinsky Institute of Organic Chemistry, Russian Academy of Sciences, Moscow, Russian Federation; 6 Laboratory of Plant Polysaccharides, N.D. Zelinsky Institute of Organic Chemistry, Russian Academy of Sciences, Moscow, Russian Federation; 7 Scottish Association for Marine Sciences, Oban, Argyll, Scotland, United Kingdom; 8 S.S. Annunziata Hospital, School of Medicine, University G. D'Annunzio, Chieti, Italy; University of Bergen, Norway

## Abstract

Sulfated polysaccharides from *Laminaria saccharina* (new name: *Saccharina latissima*) brown seaweed show promising activity for the treatment of inflammation, thrombosis, and cancer; yet the molecular mechanisms underlying these properties remain poorly understood. The aim of this work was to characterize, using *in vitro* and *in vivo* strategies, the anti-inflammatory, anti-coagulant, anti-angiogenic, and anti-tumor activities of two main sulfated polysaccharide fractions obtained from *L. saccharina: a)* **L.s.-1.0** fraction mainly consisting of O-sulfated mannoglucuronofucans and *b*) **L.s.-1.25** fraction mainly composed of sulfated fucans. Both fractions inhibited leukocyte recruitment in a model of inflammation in rats, although **L.s.-1.25** appeared to be more active than **L.s.-1.0**. Also, these fractions inhibited neutrophil adhesion to platelets under flow. Only fraction **L.s.-1.25**, but not **L.s.-1.0**, displayed anticoagulant activity as measured by the activated partial thromboplastin time. Investigation of these fractions in angiogenesis settings revealed that only **L.s.-1.25** strongly inhibited fetal bovine serum (FBS) induced *in vitro* tubulogenesis. This effect correlated with a reduction in plasminogen activator inhibitor-1 (PAI-1) levels in **L.s.-1.25**-treated endothelial cells. Furthermore, only parent sulfated polysaccharides from *L. saccharina* (**L.s.-P**) and its fraction **L.s.-1.25** were powerful inhibitors of basic fibroblast growth factor (bFGF) induced pathways. Consistently, the **L.s.-1.25** fraction as well as **L.s.-P** successfully interfered with fibroblast binding to human bFGF. The incorporation of **L.s.-P** or **L.s.-1.25**, but not **L.s.-1.0** into Matrigel plugs containing melanoma cells induced a significant reduction in hemoglobin content as well in the frequency of tumor-associated blood vessels. Moreover, *i.p.* administrations of **L.s.-1.25**, as well as **L.s.-P**, but not **L.s.-1.0**, resulted in a significant reduction of tumor growth when inoculated into syngeneic mice. Finally, **L.s.-1.25** markedly inhibited breast cancer cell adhesion to human platelet-coated surfaces. Thus, sulfated fucans are mainly responsible for the anti-inflammatory, anticoagulant, antiangiogenic, and antitumor activities of sulfated polysaccharides from *L. saccharina* brown seaweed.

## Introduction

Fucoidans represent an intriguing class of fucose-enriched sulfated polysaccharides found in the extracellular matrix of brown algae. These polysaccharides have been tested in a vast array of experimental models showing anti-coagulant, anti-tumor, immunomodulatory, anti-inflammatory, and anti-complement properties [1]–[6]. Detailed chemical structures of fucoidans depend primarily on the algal species used as source of polysaccharides [3], [6], [7]. However, even a sulfated polysaccharide isolated from a given species of brown algae may be a mixture of structurally different polymers. Thus, in spite of increasing efforts, the structure-activity relationship (SAR) of fucoidans is still an unresolved issue. Recently we have investigated the anti-inflammatory, anti-coagulant, anti-angiogenic, and anti-adhesive activities of nine different fucoidans isolated from *Laminaria saccharina* (renamed as *Saccharina latissima* [8]) *L. digitata*, *Cladosiphon okamuranus, Fucus evanescens*, *F. vesiculosus, F. serratus*, *F. distichus*, *F. spiralis*, and *Ascophyllum nodosum* as pool samples [2], [9]. We found that the different profiles of biological activities exhibited by these polysaccharides depend on variations of their structural features. Interestingly, among the most active compounds studied, those extracted from *L. saccharina* have been characterized by their prominent anti-angiogenic and anti-coagulant activities *in vitro* as well as their ability to block selectin-mediated inflammation *in vivo*.

**Figure 1:**
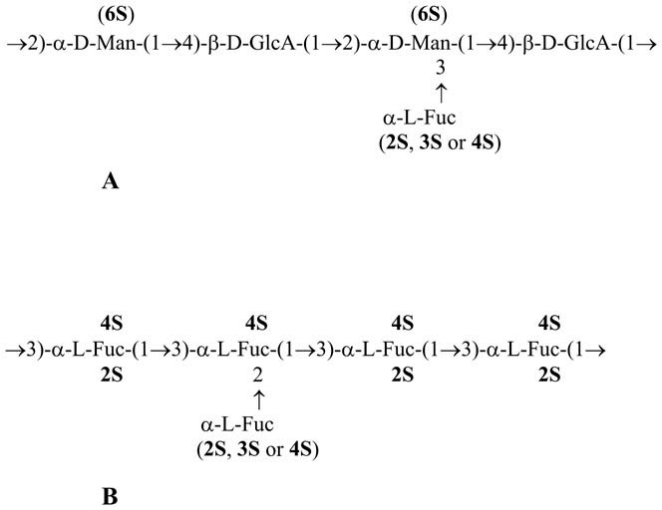
Putative structures of the main components of the fractions L.s.-1.0 (A) and L.s.-1.25 (B) obtained from the total sulfated polysaccharide preparation L.s.-P extracted from *L. saccharina*.

Here we investigated the biological profiles of two main components of the parent sulfated polysaccharide extracted from L. saccharina (**L.s.-P**). Serial fractionation by ion-exchange chromatography produced two main fractions differing in the monosaccharide composition and sulfate content [10]. The first fraction eluted from DEAE-Sephacel by 1.0 M NaCl (**L.s.-1.0**) consisted mainly of sulfated fucomannoglucuronan (Figure 1A) and not of fucoidan-related polysaccharides. The second fraction eluted by 1.25 M NaCl (**L.s.-1.25**) consisted mainly of sulfated fucans (Figure 1B), which structurally corresponds to the previously reported fucoidan from L. saccharina [11]. The presence of fucomannoglucuronan component in **L.s.-P** was surprising as this component was not clearly visible in the NMR spectra of **L.s.-P** [11]. To determine how **L.s.-1.0** and **L.s.-1.25** account for the biology activities of **L.s.-P**, we performed comparative studies of these purified fractions in terms of anti-inflammatory, anti-coagulant, anti-angiogenic, anti-tumor, and anti-adhesive functions. Results presented here provide for the first time, experimental evidence in vitro and in vivo showing that it is possible to dissect out the chemical structures responsible for the anti-inflammatory, anti-angiogenic, and anti-tumor activities of sulfated polysaccharides from L. saccharina.

## Materials and Methods

### Isolation and purification of sulfated polysaccharides from *L. saccharina*

The procedure for isolation of **L.s.-P** from *L. saccharina* has been described earlier [11]. Separation of **L.s.-P** into fractions **L.s.-1.0** and **L.s.-1.25** was performed by ion-exchange chromatography. Structural characterization of obtained fractions was conducted as previously reported [10]. The structures of the main polysaccharide components of fractions **L.s.-1.0** and **L.s.-1.25** are shown in Figure1. The sulfate contents of the starting polysaccharide preparation **L.s.-P** and its fractions are presented in Table 1. Potential traces of endotoxin contamination were carefully removed from fucoidan fractions by the Detoxi-Gel^TM^ endotoxin removing gel (Pierce) and tested as described [12] using a Gel Clot Limulus Test raising levels lower than 0.5 IU/mg; Cape Code).

**Table 1:** Sulfate content, anti-inflammatory and anti-coagulant activities of the polysaccharide preparations from *L. saccharina* brown seaweed.

Sample	Inflammation b			APTT, U/mg c	Sulfate content
	Dose mg/kg	Number of PMNs per rat (×10^6^)	Inhibition (% to control)		
Control^a^	-	49.1±5.5	-	-	-
**L.s.-P**	1.04.0	7.6±2.73.2±0.6	84.593.4	36.4±1.7	28.8
**L.s.-1.0**	1.04.0	16.2±1.58.4±1.3	67.082.9	6.8±0.2	16.2
**L.s.-1.25**	1.04.0	5.8±1.92.2±0.6	88.295.5	29.2±1.6	36.8

a Six animals received 0.9% (wt/vol) NaCl instead of polysaccharides.

b The anti-inflammatory activity was determined as the effect on PMN transmigration to the peritoneal cavity of rats (six animals in each group, for details see *Results*). The polysaccharide preparations were injected intravenously with a dose of 1.0 and 4.0 mg/kg of the rat weight. Data are presented as mean ± SEM. N is the number of rats in the group.

c Anticoagulant activity was measured as the APTT related to a heparin standard with an activity of 140 U/mg. Data are shown as mean ± SEM; N = 4.

### Clotting time assay

Anti-coagulant activity of polysaccharide samples was measured by means of an activated partial thromboplastin time (APTT) clotting assay according to the methods described [13]. Briefly, 80 µl of pooled human plasma from healthy donors were mixed with 20 µl of a solution containing 0–5 µg of sulfated polysaccharides in 0.9% NaCl, and the mixture was incubated for 1 min at 37°C. Then, 100 µl of a reagent containing a mixture of phospholipids and an activator were added, and the resulting mixture was incubated for 2 min at 37°C. Finally, a solution of 0.025 M CaCl_2_ (100 µl) was added to the mixture at 37°C. The time of clot formation was measured. The activity of **L.s.-P** or its fractions was expressed as heparin units/mg, using a parallel curve obtained with heparin standard (Fluka) with an activity of 140 U/mg.

### Cell cultures

All performed cell culture experiments were approved by local Institutional Review Board of the University of Chieti (Italy). With the written consent of the parents, fresh human umbilical cords were obtained from full-term births, aseptically stored in sterile saline and processed within 6 hours from partum to obtain endothelial cells. Human umbilical vein endothelial cells (HUVECs) were isolated by collagenase digestion as previously described [14]. HUVECs from passage 1 to 5 were grown on gelatin-coated dishes in 199 Medium (M199) containing 10% FBS (Gibco), supplemented with 12 U/ml heparin and 50 µg/ml bovine crude endothelial cell growth factor (ECGF) at 37°C under 5% CO_2_. MDA-MB-231 breast cancer cells were grown in Dulbecco's Modified Eagle Medium (DMEM) supplemented with 10% heat-inactivated fetal bovine serum. Fibroblasts were grown in DMEM supplemented with 10% fetal calf serum (FCS) (Gibco). B16-F10 mouse melanoma cells were cultured in DMEM essentially as described [12].

### Isolation of platelets and polymorphonuclear neutrophils (PMNs) from human blood

Blood was collected from healthy volunteers who had not received any medication for at least 2 weeks. They were selected among staff scientists of the Institute Consorzio Mario Negri Sud and donated their blood for research purposes. Written consent of donors and the approval from the institutional (Consorzio Mario Negri Sud) Review Board were obtained for these studies. All further *in vitro* assays are performed in University of Chieti following the guidelines of Institutional Ethics Committee, University of Chieti. Blood was anti-coagulated with 3.8% trisodium citrate at a 9∶1 ratio. Human platelets were prepared by differential centrifugation as described [2]. After removing the platelet-rich plasma, PMNs were isolated by dextran sedimentation followed by Ficoll–Hypaque gradient and hypotonic lysis of erythrocytes. PMNs were washed and resuspended in HEPES–Tyrode's buffer (pH 7.4) containing 129 mM NaCl, 9.9 mM NaHCO_3_, 2.8 mM KCl, 0.8 mM KH_2_PO_4_, 0.8 mM MgCl_2_.6H_2_O, 5.6 mM glucose, 10 mM HEPES and 1 mM CaCl_2_.

### Platelet monolayers

Glass cover slips were coated with 4% 3-aminopropyltriethoxysilane (APES) in acetone. 500 µl of 1 M CaCl_2_ containing 3.5×10^7^ platelets/ml were stratified on APES-coated glass-slides, and platelets were allowed to adhere for 3 h at room temperature. The density and confluence of platelet layers were examined by light microscopy.

### Flow adhesion assay

PMNs adhesion to adherent platelets was investigated in a parallel plate flow chamber under physiologic flow. Platelet-coated slides mounted in a flow chamber were placed in a thermoregulated plexiglass box maintained at 37°C by an electric heating element. The platelet surface was perfused with 5 ml of a PMN suspension (10^6^/ml in 0.1% bovine serum albumin (BSA)/DMEM) at a wall shear stress of 2 dynes/cm^2^ for 2 min followed by perfusion with a cell-free medium at a wall shear stress of 10 dyne/cm^2^ for 2 min in order to remove nonadherent PMNs. The interaction of PMNs with platelets was observed by phase contrast video microscopy with a 20X/NA objective (Olympus, Munich, Germany), and the images were continuously recorded for playback analysis (Pro-Series video camera, High Performance CCD camera, Media Cybernetics, Silver Spring, MD). Adherent PMNs were counted at the end of the perfusion in four randomized fields by using an *ad hoc* software for image analysis (Image Pro-Plus for Windows, Media Cybernetics, Silver Spring, MD) and reported as percentage ± SD, compared to control. In selected experiments, P-selectin or the β_2_-integrin adhesive function was blocked by incubating platelets or PMNs for 10 min at RT with 20 µg/ml of monoclonal antibodies, WAPS 12.2 and IB4 [15], respectively. The platelets were pre-incubated with **L.s.-P** or its fractions (100 µg/ml, 500 µl/slide) for 15 min at RT before PMNs perfusion.

### Tubulogenesis assay

The ability of **L.s.-P** and its fractions to modulate angiogenesis *in vitro* was evaluated in a capillary tube formation (tubulogenesis) assay as previously reported [2]. Briefly, chamber slides were coated with growth factor-depleted Matrigel (BD Pharmingen) for 1 h at 37°C. HUVECs resuspended in M199 containing 10% FBS were seeded on Matrigel (5×10^4^/well). **L.s.-P** or its fractions (**L.s.-1.0** and **L.s.-1.25)** were added at a final concentration of 100 µg/ml. After an incubation of 18 h at 37°C and 5% CO_2_, cultures were photographed. For each individual well, three digitalized pictures were taken from different fields. Pictures were analyzed by ImagePro Plus software (Media Cybernetics, Silver Spring, MD) and closed areas (tube-like structures) were counted. The extension of tube formation was expressed as the percentage of tubes identified on treated samples *versus* controls. The final results were pooled from at least three separate experiments.

### Plasminogen Activator Inhibitor-1 (PAI-1) antigen assay

Levels of PAI-1 antigen were measured in conditioned media (CM) from HUVECs plated on Matrigel for the tubulogenesis assay in the presence or absence of **L.s.-P** or its fractions. After 20 h of incubation, CM were collected, centrifuged and stored. Five µl of CM were used for the measurement of PAI-1 using an enzyme-linked immunosorbent assays (ELISAs) (American Diagnostica GmbH, Germany).

### *In vitro* platelet-tumor cell adhesion assay

For the tumor cell adhesion assay, platelet-coated surfaces were generated as described [2]. Briefly, 3×10^7^ platelets resuspended in HEPES Tyrode's buffer were added to flat-bottomed plastic microtiter wells. The plate was incubated for 1 h at RT. Afterwards, the same volume of a HEPES Tyrode's buffer enriched with 2 mM CaCl_2_ was added to platelets. Then, plates were left at 4°C overnight, non-adherent platelets were removed by washing with 1% BSA solution in phosphate buffered saline (PBS), and the adhered platelets were then incubated for 1 h at 37°C with 200 µl of 1% BSA solution in PBS in order to block "free-adhesive" sites on the plastic surface. Tumor cells were detached and re-suspended in PBS enriched with CaCl_2_ and MgCl_2_. After counting, 1×10^5^ cells were added to each platelet-coated well in the presence or absence of 100 µg/ml of **L.s.-P, L.s.-1.0** or **L.s.-1.25**, and incubated at 37°C for 1 h. The plates were then washed twice and fixed with methanol. Adherent cells were stained by haematoxylin in order to make nuclei visible. Different fields were photographed, and the number of adherent cells was evaluated by counting.

### *In vivo* angiogenesis and tumor growth

This work has been approved by the Institutional Ethics Committee and Review Board of the Institute of Biology and Experimental Medicine (IBYME; Buenos-Aires, Argentina) of National Council of Scientific and Technical Investigations (CONICET) (approval #A5072-01 014/1) and conducted in accordance with the “Principles of laboratory animal care” (NIH Publication no. 85-23, revised 1985). C57BL/6 (B6) mice were injected with 500 µl Matrigel containing 1×10^5^ B16-F10 cells and PBS as control or 100 µg of **L.s.-P**, **L.s.-1.0** or **L.s.-1.25**. Matrigel plugs were removed after 7 days and the extent of new blood vessels formation was evaluated by hemoglobin content. CD34^+^ cells present in Matrigel plugs were evaluated by flow cytometry using a PE-conjugated anti-CD34 monoclonal antibody (eBiosciences) on a FACSAria^TM^ (BD Biosciences). For confocal microscopy analysis, mice were anesthetized and cardiac-perfused with PBS and 4% paraformaldehyde and tissues were embedded in frozen Optimum cutting Temperature (OCT) medium. Tumors were cut (30 µm) in a cryostat and were frozen at -70°C. For immunohistochemical staining, section samples were dried at RT and fixed in acetone for 10 min at −20°C. After air-drying for 5 min, the nonspecific binding sites were blocked by incubation for 1 h at RT in 10% normal rat serum. Sections were stained with the rat anti-mouse PECAM-1/CD31 (clone MEC13.3; BD) at 1∶200 dilution overnight at 4°C followed by goat anti-rat AlexaFluor 596 conjugate (Cell Signaling), 1∶1000 for 1 h at RT. The slides were washed and mounted in Fluormount mounting medium, and immunofluorescence images were collected on a Nikon E800 confocal microscope. Microvessel density (MVD) was determined by counting the number of microvessels in five randomly selected fields (200X) per tumor.

For long-term *in vivo* experiments, **L.s.-P** or its fractions were administered *i.p.* every 3 days, starting from 24 h after Matrigel injection. The animals were examined daily, and the body weight was recorded twice weekly. Details on doses and schedules of treatments are given in the relevant figure legends. Matrigel plugs were removed on the day 21 post-implantation, weighed, photographed and processed. Parameters of angiogenesis were evaluated as above.

### Rat peritoneal inflammation model

This work has been approved by the Institutional Ethics Committee and Review Board of the V.N. Orekhovich Research Institute of Biomedical Chemistry, Russian Academy of Medical Sciences (approval #7-2007 of June 11, 2007). A rat model of acute peritonitis was used as described [2] with some modifications. Nine ml peptone (8.0% solution in 0.9% NaCl) were injected *i.p.* into female Wistar rats (about 250 g, six animals in each group) under ether anesthesia. Solutions of tested **L.s.**-polysaccharides (with a dose of 1.0 and 4.0 mg/kg of the rat weight) in sterile 0.9% NaCl (0.3 ml) were injected into the femoral veins of the animals 15 min after peptone injection. The same volume of 0.9% NaCl was injected to control animals. After 3 h, animals were anesthetized and sacrificed, and their peritoneal cavities were washed with 30 ml of PBS containing 60 U/ml heparin, 0.02% ethylenediaminetetraacetic acid (EDTA), and 0.03% bovine serum. The cell number in the lavage was determined, and the cell suspension was concentrated by centrifugation at 400×g for 10 min. After 1∶1 dilution with bovine serum, the smears were prepared and stained by the Pappenheim method. The number of PMNs was determined in two parallel smears, each containing 600 cells.

### Statistical Analysis

Statistical significance was determined with the Student's *t* test. *P* values less than 0.05 were considered as significant. The Kaplan-Meier and one-way ANOVA analysis was used to establish statistical significance for *in vivo* experiments.

## Results

### Chemical structure of fucoidan fractions from brown algae of *L. saccharina*

Among the polysaccharides of nine different brown algal species, those derived from L. saccharina, **L.s.-P**, were found to be the most potent in terms of anti-inflammatory, anti-coagulant, anti-adhesive and anti-angiogenic activities [2], [9]. To evaluate the chemical determinants of **L.s.-P** responsible for the distinct biological activities, we proceeded to its fractionation by using ion-exchange chromatography [10]. Surprisingly, two major fractions related to different types of O-sulfated polysaccharides were obtained: mannoglucuronofucans (fraction **L.s.-1.0**) and sulfated fucoidans (fraction **L.s.-1.25**), which consist mainly of fucose units and have a higher degree of sulfation (Figure 1 and Table 1). Figure 1 shows the essential structural motifs of the polysaccharides from **L.s.-1.0** and **L.s.-1.25**, whose monosaccharide units have partial sulfations at the indicated sites [10]. To compare the biological profiles of the parent non-fractionated polysaccharide **L.s.-P** and its fractions **L.s.-1.0** and **L.s.-1.25**, we examined their biological properties in terms of anti-coagulant, anti-inflammatory, anti-adhesive, anti-angiogenic and anti-tumor activities using different bioassays [2].

### Anticoagulant activities of fucoidan fractions from brown algae of *L. saccharina*

While studying the activities of fucoidans from eleven brown algae species, we have shown that the total fucoidan from *L. saccharina* (**L.s.-P**) was the most active anticoagulant in the APTT test [2], [8]. Comparative studies of anticoagulant activities of **L.s.-P**, fractions **L.s-1.0** and **L.s-1.25** demonstrated that the **L.s.-1.25** fraction exhibits higher activity than the **L.s.-1.0**, probably due to structural differences in sulfate contents (Table 1). Notably, the starting preparation **L.s.-P** was slightly more active than **L.s.-1.25** in spite of its lower sulfate content. This fact may be explained by the synergistic action of both fractions **L.s.-1.0** and **L.s.-1.25** present in combination or by the loss of more active minor components of **L.s.-P** during the fractionation.

### Anti-inflammatory activities of fucoidan fractions from brown algae of *L. saccharina*

We have previously shown that fucoidans from *L. saccharina* interfere with L- and P-selectins, thus promoting a decrease in PMN transmigration to the abdominal cavity and blocking the induction of acute peritonitis [16]. In the present study, by using the same acute peritonitis rat model, we explored the effect of the *L. saccharina*-derived fractions **L.s.-1.0** and **L.s.-1.25** on PMN transmigration. Our results demonstrate that both the **L.s.-1.0** and **L.s.-1.25** fractions at a dose of 1 mg/kg inhibited, although at different extents, PMN influx into the peritoneal cavity (Table 1). As compared to the control, **L.s.-1.25** significantly inhibited the PMN influx (89.3%, *P*<0.05) with an activity similar to that of **L.s.-P**. However, although statistically significant (*P*<0.05), the effect of **L.s.-1.0** was less pronounced (68%).

### Effects of fucoidan fractions from brown algae of *L. saccharina* on PMN adhesion to platelets under flow conditions

**Figure 2:**
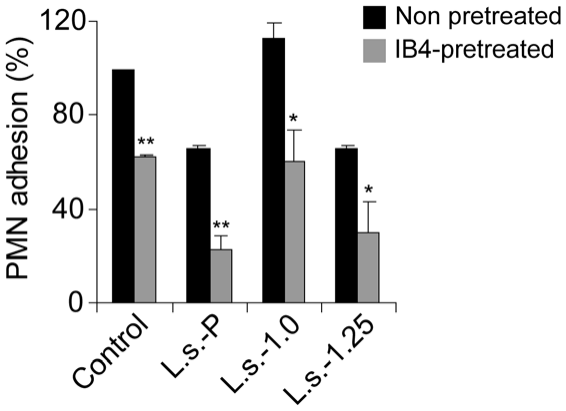
Effect of L.s.-P and its fractions L.s.-1.0 and L.s.-1.25 on PMN adhesion to platelet-coated surface under flow conditions. Polysaccharide fractions **L.s.-1.0** and **L.s.-1.25** or a parent mixture **L.s.-P** were added at a final concentration of 100 µg/ml to a platelet-coated surface and incubated for 10 min at RT. The same concentration of each compound was also added to non-treated (filled bars) or IB4-pretreated (grey bars) PMN suspensions before addition to platelets. Under the flow conditions, migration of PMN was monitored and photographed. Analysis was performed by counting the number of attached PMN per field in at least 4 different fields. The mean percentage ± SEM with respect to control of at least three independent experiments is represented. *P<0.05; **P<0.01.

Blocking of P-selectins represents one of the mechanisms underlying the efficacy of fucoidans in reducing PMNs transmigration *in vivo*, as suggested by the studies on PMN adhesion to platelet-coated surfaces under flow conditions [2]. In this assay, blockage of either P-selectin or β_2_-integrin by using the specific antibodies WAPS (not shown) or IB4 prevented PMN adhesion either completely or partially. To confirm the ability of fucoidan fractions to block such an interaction, 100 µg/ml of each polysaccharide was pre-incubated with platelets before PMN infusion. As shown in Figure 2, addition of **L.s.-1.25**, as well as **L.s.-P**, was able to reduce the number of adherent PMN down to 30%, whereas **L.s.-1.0** was not effective in modulating this effect. To gain further mechanistic insights, IB4-pretreated PMNs were also assayed (Figure 2, opened bars). In this case, the inhibitory activity of **L.s.-1.25** was markedly augmented (almost 60% of inhibition) when β_2_-integrin was blocked. These results suggest that similar to the non-fractionated **L.s.-P** polysaccharides [2], [15], **L.s.-1.25** fraction inhibits PMN adhesion to platelets through a P-selectin-dependent mechanism.

### Effects of fucoidan fractions from brown algae of *L. saccharina on* FBS-induced HUVEC capillary-like structures formation *in vitro*

**Figure 3:**
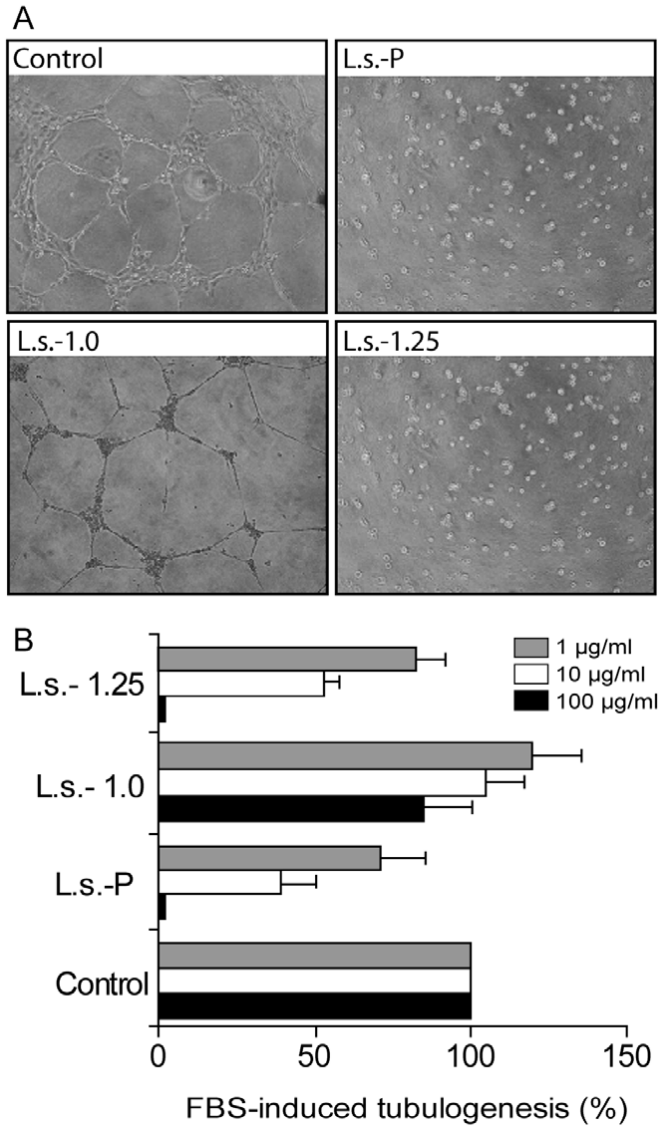
Selective inhibitory effects of L.s.-P and its fractions L.s.-1.0 and L.s.-1.25 on FBS-induced HUVEC tubulogenesis. (**A**) Representative photographs of HUVEC cultured on Matrigel in the presence of FBS along with 100 µg/ml of indicated parent fucoidan or purified fractions. (**B**) Quantitative analysis of tube-like structures *in vitro* using three different polysaccharide concentrations. Analysis was obtained by counting closed areas (tubes) in at least four different fields. Data are collected from at least three independent experiments. All data were expressed as the percentage of tubes/cm^2^ of treated cells *vs* control: filled, open and grey bars indicate the effect induced by 100, 10 or 1 µg/ml polysaccharides respectively.

As previously established, the non-fractionated fucoidan preparation **L.s.-P** from *L. saccharina* has a pronounced anti-angiogenic activity demonstrated by its ability to reduce FBS-induced HUVEC tubulogenesis [2]. In the present study, we aimed to identify the fractions retaining these properties. As shown in Figure 3, addition of 100 µg/ml **L.s.-1.25** to HUVEC suspensions before plating completely prevented (99% of inhibition; *P*<0.001) tube formation, showing, a comparable inhibitory effect to that induced by **L.s.-P**, whereas **L.s.-1.0** displayed no significant biological activity. Remarkably, endothelial cell morphogenesis was still significantly inhibited when lower concentrations of **L.s.-1.25** were used (Figure 3B). As our previous results, [17] we measured the levels of PAI-1 in HUVEC embedded in Matrigel cultured in the presence or absence of fucoidan fractions. The levels of PAI-1 were markedly reduced (almost 60% and 47% respectively) when **L.s.-P** and **L.s.-1.25** were added to HUVEC cultures while **L.s.-1.0,** which was not capable of influencing HUVEC tubulogenesis, did not affect PAI-1 release from these cells (data not shown).

### Effects of fucoidan fractions from brown algae of *L. saccharina on* bFGF-mediated angiogenesis

**Figure 4:**
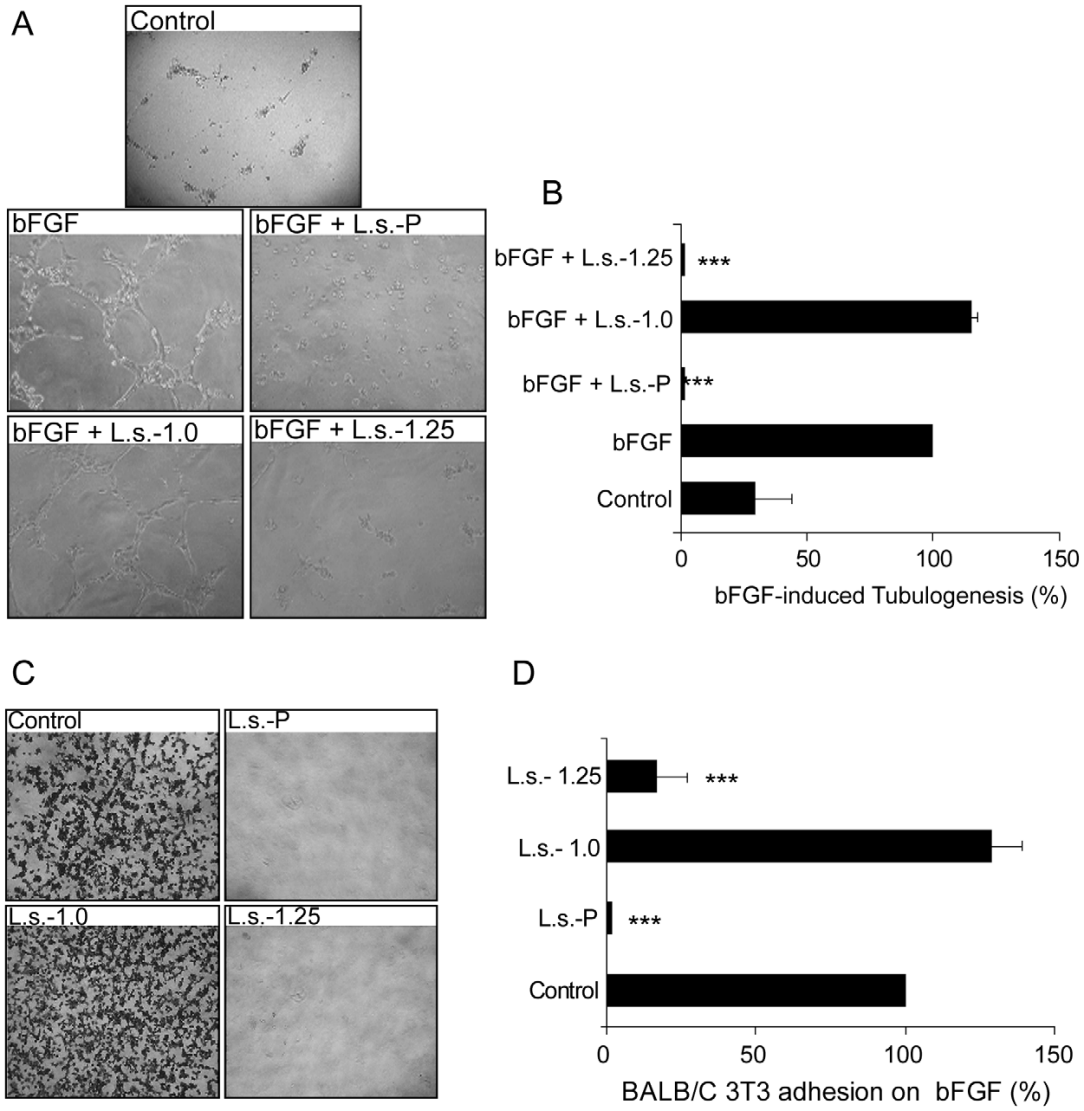
(A-B) Specific effect of L.s.-P and its fractions L.s.-1.0 and L.s.-1.25 on the inhibition of bFGF-induced HUVEC tubulogenesis. (A) Representative photographs of HUVEC cultured on Matrigel in the presence of bFGF along with 100 µg/ml of the indicated polysaccharide preparations. (B) Quantitative analysis of tube-like structures. All data were expressed as the percentage of tubes/cm^2^ *vs* control (bFGF). (C,D) Specific effect of **L.s.-P** and its fractions **L.s.-1.0** and **L.s.-1.25** on the inhibition of Balb/c 3T3 adhesion to bFGF. (C) Effects of **L.s.-P** and **L.s.-1.25** on fibroblast cell adhesion to bFGF. Representative images of fibroblast cell adhesion to purified bFGF are shown. The images are representative of three independent experiments. Quantification was performed by counting adhered cells of at least three different fields. Results are expressed as percentage of the treated sample with respect to control (D). ***P<0.001.

bFGF is an essential mediator capable of regulating angiogenesis [18]. We examined whether the parent fucoidan from *L. saccharina* or its fractions could selectively modulate bFGF-mediated events. We initially investigated the effect of such polysaccharides by targeting bFGF-induced HUVEC tubulogenesis For such assay, each polysaccharide and bFGF were added before plating HUVEC onto Matrigel. As shown in Figure 4A–B, addition of 100 µg/ml parent fucoidan from *L. saccharina* (**L.s.-P**) or the **L.s.-1.25** fraction blocked (99% inhibition, *P*<0.001) bFGF-induced HUVEC tube formation, while **L.s.-1.0** had no effect.

The particular chemical structure and conformation of fucoidans suggested the possibility that these polysaccharides may directly interfere with the binding of growth factors to their specific cognate receptors [3], [19]. Therefore, we examined the ability of each fraction to interfere with bFGF-receptor interactions on BALB/c 3T3 fibroblasts [20]. Parent sulfated polysaccharides (**L.s.-P**) or the above-mentioned fractions were added separately at final concentrations of 100 µg/ml to fibroblasts suspensions on bFGF-coated plates. As shown in Figure 4C–D, adhesion of fibroblasts to bFGF was completely abolished by **L.s.-P** or **L.s.-1.25** preparations, but not by **L.s.-1.0**.

### Effects of fucoidan fractions from brown algae of *L. saccharina* on *in vivo* tumor growth and angiogenesis

**Figure 5:**
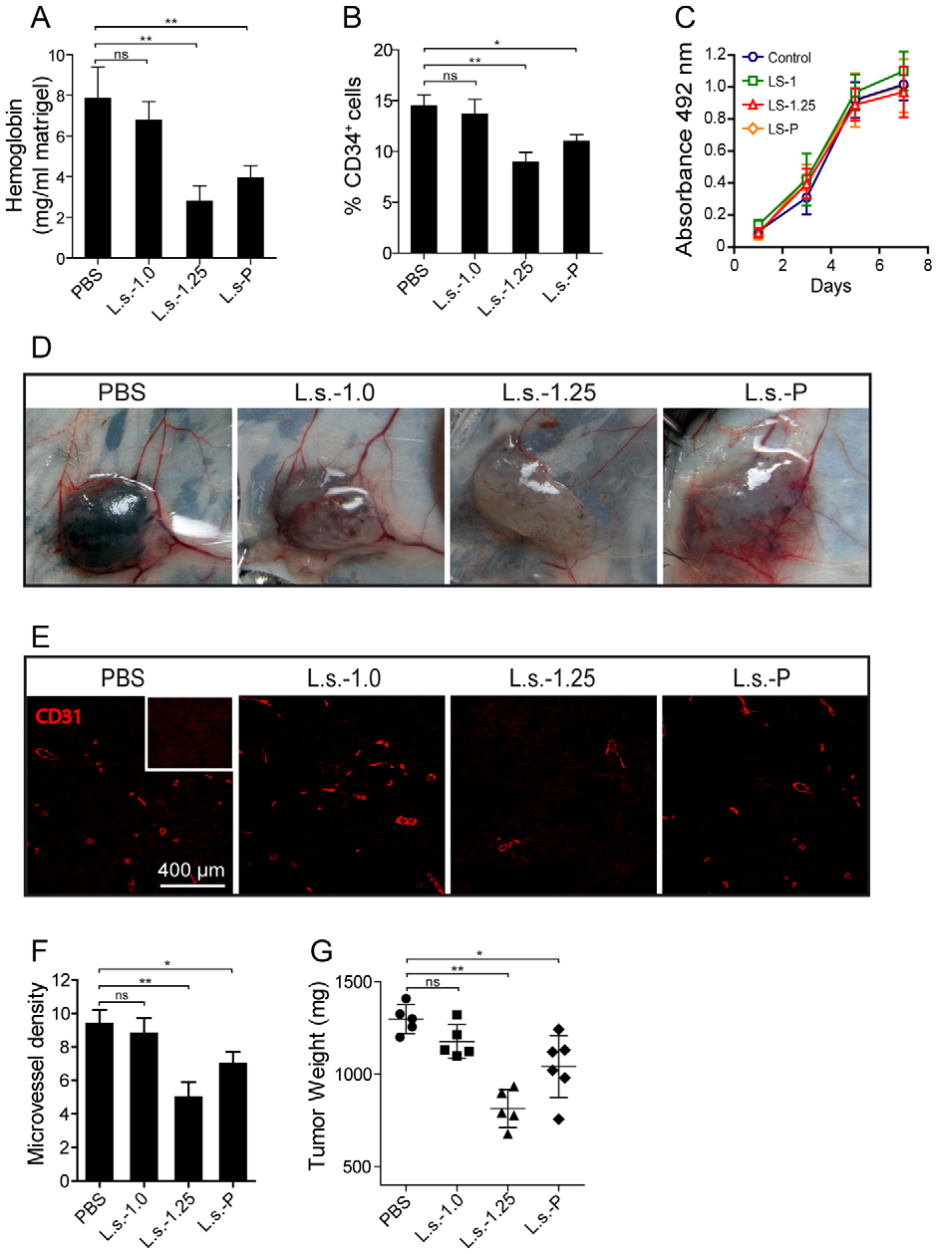
Effect of polysaccharide preparations on tumor growth and angiogenesis *in vivo*. C57BL/6 (B6) mice were injected with 500 µl of Matrigel containing 1×10^5^ B16-F10 cells in PBS or 100 µg of a non-fractionated polysaccharide mixture **L.s.-P** or its fractions **L.s.-1.0** and **L.s.-1.25**. After 6–7 days, tumors were removed and hemoglobin content was evaluated by using the Drabkin colorimetric method. Results are expressed as the amount of hemoglobin (mg)/Matrigel weight (mg) (**A**) (**P<0.01). (**B**) Flow cytometry analysis of the frequency of CD34^+^ endothelial cells on Matrigel plugs embedded with B16 melanoma cells. (**P<0.01) (**C**) *In vitro* cell growth of B16 melanoma cells exposed to 100 µg/ml of **L.s.-P** or its fractions **L.s.-1.0** and **L.s.-1.25**. Data are the mean ± SEM of three independent experiments. (**D**) B6 mice were injected with 500 µl Matrigel containing 1×10^5^ B16-F10 cells. **L.s.-P** or its fractions **L.s.-1.0** and **L.s.-1.25** were injected *i.p.* at doses of 50 mg/kg every 3 days and compared to control (PBS). Tumors were removed on day 21 post-implantation, photographed (**D**) and analyzed for CD31^+^ associated blood vessels (**E**), microvessel density (**F**) and weight (**G**). (*P<0.05; **P<0.01).

Given their ability to influence endothelial cell biology *in vitro,* we further studied the effects of the total sulfated polysaccharide preparation from *L. saccharina* (**L.s.-P**) and its purified fractions on tumor growth and tumor-related angiogenesis *in vivo*. For this, B6 mice were injected *s.c.* with Matrigel plugs enriched with melanoma B16-F10 cells as described [21] and 100 µg of **L.s.-P** or the same amount of each fraction. Six to seven days after implantation, Matrigel plugs were collected to determine hemoglobin content and the frequency of CD34^+^ endothelial cells, which represent reliable measurements of neovascularization. As compared to control Matrigel plugs, those containing **L.s.-P** or **L.s.-1.25**, but not **L.s.-1.0** fraction, showed a significant reduction in the levels of hemoglobin content (Figure 5A) and the frequency of CD34^+^ cells (Figure 5B), suggesting important effects on angiogenesis. However, *in vitro* exposure to **L.s.-P**, **L.s.-1.0** or **L.s.-1.25** did not affect melanoma cell proliferation (Figure 5C), ruling out a direct effect of these polysaccharides on tumor cell growth.

Given their pronounced anti-angiogenic effects, we further explored the effect of therapeutic *i.p.* injection of different polysaccharides in mice bearing B16-F10 melanoma Matrigel-plugs. To this end *i.p.* treatments (50 mg/kg) were performed twice a week starting from the day following Matrigel sponge implantation. A significant reduction of tumor angiogenesis and microvessel density was observed only in mice receiving **L.s.-P** or **L.s.-1.25**, but not in mice treated with **L.s.-1.0** (Figure 5D–F). These anti-angiogenic effects were accompanied by a significant reduction in tumor weight in **L.s.-P-** or **L.s.-1.25-**treated mice (Figure 5G). Importantly, the mean weight of animals treated with **L.s.-P** or its fractions did not differ from that of control animals, and no significant histopathologic changes were observed in sections of liver, spleen and lung of treated versus untreated mice (data not shown), suggesting that fucoidans or their purified fractions have no substantial toxic effects at least at the concentrations tested.

### Effect of fucoidan fractions from brown algae of *L. saccharina* on breast cancer cell adhesion to human platelets

**Figure 6:**
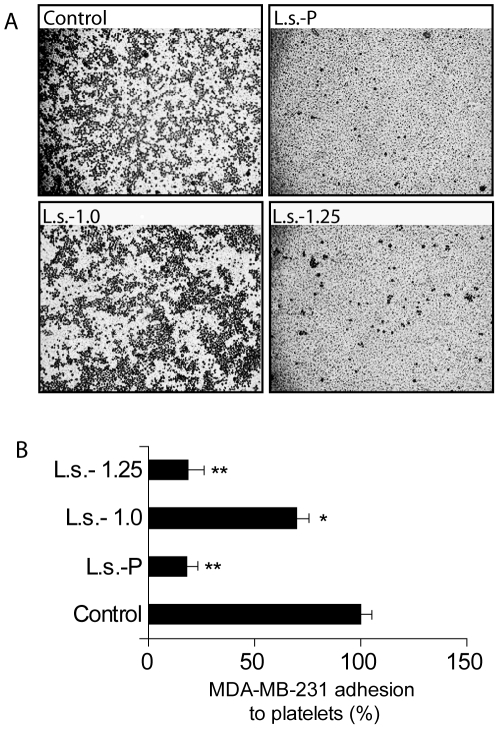
Selective effects of L.s.-P and its fractions L.s.-1.0 and L.s.-1.25 on breast cancer cell adhesion to human platelets. (A) MDA-MB-231 breast cancer cells were pre-incubated with selected polysaccharide preparations prior to exposure to human platelet-coated plates. The images are representative of three independent experiments. (B) Quantitative analysis of cell adhesion was performed by counting the number of tumor cells adhered to at least three different fields. Results are expressed as mean percentage ± SEM of the treated samples versus control. *P<0.05; **P<0.01.

We have recently demonstrated that the pool of sulfated polysaccharides from *L. saccharina* efficiently blocks the adhesion of a highly metastatic tumor cell line to human platelets [2], suggesting that this compound could also be a promising inhibitor of one of the earliest processes favouring tumor metastasis [22]. Here we examined the ability of **L.s.-1.0** and **L.s.-1.25** fractions to abrogate breast cancer cell adhesion to platelet-coated surfaces. Each preparation was added at a final concentration of 100 µg/ml to MDA-MB-231 breast cancer cell suspension before plating the cells, which were left to adhere to platelet-coated surfaces for 1 h. As shown in Figure 6A–B, the non-fractionated preparation **L.s.-P** inhibited tumor cell adhesion to platelets by 80%. Interestingly, both isolated fractions **L.s.-1.0** and **L.s.-1.25** significantly suppressed these heterotypic interactions, but **L.s.-1.25** fraction was much more active than **L.s.-1.0** (80%; *P*<0.003 reduction *vs* 30% reduction; *P*<0.05). These results demonstrate that the ability of **L.s.-P** to inhibit heterotypic cell adhesion between tumors and human platelets is mainly associates with the effect of the **L.s.-1.25** fraction.

## Discussion

Data from over 700 studies indicate that fucoidans from brown seaweeds are endowed with great potential as safe nutritional and possible therapeutic agents for a wide variety of health complaints. These sulfated biopolymers are represented by very complex and structurally heterogeneous polysaccharides, which mainly contain substantial percentages of L-fucose and sulfate ester groups together with other types of monosaccharides, uronic acid, acetyl groups, and other non-carbohydrate substituents [3], [6]. Although such properties make very difficult the detailed structural elucidation of fucoidans, increasing efforts are devoted to the identification of a novel structure-activity relationship. It has been shown that the chemical structures of fucoidans vary from species to species and different extraction and/or purification methods could substantially change the bioactivity of fucoidans by modifying the parent chemical structures [3], [10], [15]. These data have reinforced the notion that several chemical features of fucoidans can profoundly influence their bioactivities. In accordance, we have recently reported that, among fucoidans derived from nine different brown algal species, the polysaccharide isolated from *L. saccharina* (renamed as *Saccharina latissima*) represents the most powerful inhibitor endowed with anti-inflammatory, anti-coagulant, anti-adhesive, and anti-angiogenic properties [2].

The aim of the present study was to delineate chemical motifs of the fucoidan structure of *L. saccharina*, which might reflect improved biological activities of these polysaccharides, mainly those related to cancer biology. Using ion-exchange chromatography the parent polysaccharide **L.s.-P** was separated into two main fractions related to two different types of sulfated polysaccharides: fucomannoglucuronan (fraction **L.s.-1.0**) and fucan (fraction **L.s.-1.25**) [10]. The latter fraction consists mainly of fucose units and has a higher degree of sulfation (Figure 1). To compare the biological activities of the parent polysaccharide **L.s.-P** with those of its derived fractions **L.s.-1.0** and **L.s.-1.25**, we evaluated their anti-coagulant, anti-inflammatory, anti-adhesive, anti-angiogenic, and anti-tumor effects.

Fucoidans, like heparin, represent promising anti-coagulant agents, being able to affect different coagulation pathways [23], [24]. As a result, in the past years, fucoidans have been recommended as substitutes for heparin in the therapy of thrombosis [23], thus avoiding the risk of “prion” contamination affecting mammalian-derived compounds. Due to the clinical relevance of these therapies, several attempts have been made to enhance the anti-coagulant activity of fucoidans. In this respect, it has been suggested that the enhanced activity correlates with some chemical characteristics, such as the sugar composition (fucose, galactose, etc.), the sulfate content and position and the molecular weight [3]. A similar relationship was confirmed by studies on sulfated fucans isolated from marine invertebrates (the jelly coat of sea urchin eggs and the body wall of sea cucumbers), which possess, unlike brown algal fucoidans, a regular structure [25]. In addition to a series of relevant features, we found that the presence of the 2-O-α-D-glucuronyl branch may decrease the anti-coagulant properties of fucoidans, as in the case of a fucoidan from the brown algae *C. okamuranus* [2]. Interestingly, in this study only one of the isolated fractions, namely, **L.s.-1.25**, could successfully prolong the APTT time in the human plasma coagulation test, as shown in Table 1. This effect could be associated to the higher sulfate content present in **L.s.-1.25**.

Another interesting feature of fucoidans is represented by their anti-angiogenic and anti-tumoral activities. Inhibiting or normalizing the formation of tumor vascular networks represents a promising strategy for controlling several malignancies [26]. More than 75 inhibitors of angiogenesis have been included in clinical trials, but only few of them were found to be efficient as single-therapy for advanced tumors [27]. This lack of success could be explained by the presence of alternative mechanisms that may compensate for the blockade of canonical angiogenic pathways, mainly those triggered by the vascular endothelial growth factor (VEGF) and the bFGF. Moreover, it has been proposed that some anti-angiogenic agents may augment tumor hypoxia by inducing endothelial cell apoptosis, thus facilitating tumor progression and metastasis [27]. These clinical observations prompted the identification of novel anti-angiogenic agents capable of promoting vascular remodeling without drastically altering endothelial cell survival. In this respect, fucoidans from *L. saccharina* represent promising candidates for novel anti-tumor therapies, as it is a non-toxic naturally occurring compound, which can be potentially produced in a large scale and could influence different angiogenic and metastatic-related processes [2]. Moreover, as mentioned above, it is highly feasible to improve fucoidan biological activity by the separation of more active fractions and even chemical manipulation. For instance, it has been shown that an additional sulfation can increase the anti-angiogenic and anti-tumoral activities of the fucoidan from *F. vesiculosus* [28].

Here we demonstrate that only one fraction (**L.s.-1.25**) derived from parent polysaccharide **L.s.-P**, which is composed of O-sulfated fucose units, represents an inhibitor of *in vitro* HUVEC angiogenesis, as shown by reduction of FBS-induced tubulogenesis (Figure 3) and PAI-1 levels (data not shown) Although the mechanisms underlying the inhibition of *in vitro* tubulogenesis are not fully understood, interference of fucoidans with the activities of the relevant angiogenic growth factors has been proposed [3], [28], [29]. In this regard, a recent study has documented the ability of fucoidans to inhibit VEGF-induced angiogenesis and tumor neovascularization *in vivo,* likely through modulation of cell surface neuropilins (NRP1 or NRP2) and other VEGF receptors [19]. In addition, other studies reported that both the sulfation and size of polysaccharides are critical for NRP1 internalization [30]. Illustrating this concept, a low-molecular-weight (5,000 Da) fucoidan did not reduce the cell surface NRP1 levels in endothelial cells. These results are consistent with the critical role of glycosylation in the regulation of inflammation and angiogenesis [31], [32].

Supporting these findings, here we demonstrated that a mixture of sulfated polysaccharides from *L. saccharina* (**L.s.-P**) or its fraction **L.s.-1.25** can inhibit bFGF-induced tubulogenesis (Figure 4A–B) by interfering with the binding of this growth factor to its cell surface receptors and heparin sulfate proteoglycans [20]. Moreover, preliminary data confirmed the ability of both **L.s.-P** and **L.s.-1.25** to neutralize the effect of other important mitogenic factors, such as the platelet-derived growth factor (PDGF), as suggested by tubulogenesis experiments of PDGF-induced transfected PAECs (not shown). Collectively, these data indicated that fucoidan fraction **L.s.-1.25** obtained from *L. saccharina* could serve as a specific inhibitor of angiogenesis *in vitro*. This concept is strengthened by the finding that fucoidan fractions act as a potent P-selectin inhibitor [2]. P-selectin, a critical cell adhesion molecule expressed either by platelets or activated endothelium, is directly involved in the development of inflammatory processes [8], but can also contribute to angiogenesis by promoting endothelial cell migration [33]. Moreover, P-selectin also plays a key role in the initial process of tumor cell adhesion to vascular endothelial cells and in heterotypic interactions between activated platelets and tumor cells during metastasis [22]. Therefore, P-selectin blockade is not only envisaged as a promising anti-inflammatory strategy but also represents a potential approach to suppress angiogenesis and metastasis by inhibiting endothelial cell migration and disrupting the formation of tumor cell-platelet emboli complexes. Interestingly, we found that the **L.s.-1.25** fraction markedly inhibits adhesion of the MDA-MB-231 breast cancer cell to human platelets. As previously described, the contribution of other adhesion molecules is assured in such a phenomenon, since the single use of anti-P selectin WAPS monoclonal antibody was not sufficient to impair cell-cell adhesion [2]. Hence, the fucoidan fraction **L.s.-1.25** might potentially neutralize other important players involved in tumor cell adhesion including thrombospondin or integrins [2].

The effectiveness of sulfated polysaccharides from *L. saccharina* on angiogenesis was also evaluated *in vivo*. Our results show that non-fractionated polysaccharides **L.s.-P** and the fucoidan fraction **L.s.-1.25**, but not the mannoglucuronofucan fraction **L.s.-1.0,** markedly reduce tumor-associated angiogenesis and growth of melanoma cells *in vivo*. This finding was not related to an effect of polysaccharide samples on the intrinsic proliferative properties of melanoma cells, since they had a similar growth rate as shown by the MTT assay following treatment with either control PBS, **L.s.-P,** or its fractions **L.s.-1.25** or **L.s.-1.0**. Moreover, we found a significant reduction of tumor-associated blood vessels and tumor growth when **L.s.-P** or **L.s.-1.25** was administered *in vivo* in tumor-bearing mice. Importantly, we could find no signs of toxicity associated to changes in body weight or alterations in the histology of different organs as previously demonstrated in other settings [34]–[36]. In this regard, several studies showed that prolonged administration of fucoidans is not toxic with regards to body weight variations, haematological disorders or histopathologic alterations [34]–[36].

In spite of the fact that brown seaweed fucoidans have been the subject of many different biomedical investigations, their structures need to be characterized in more detail. In previous report [2], [9], we could not detect minor sulfated fucoglucuronan, fucoglucuronomannan, and fucogalactan in fucoidans from *L. saccharina*. These components were not easily detectable in total fucoidan extracts and became visible only after the fractionation by ion-exchange chromatography, as described here. Thus, the use of commercially available crude fucoidan samples in the investigation of structure-activity relationships is not recommended due to structural uncertainty of such polysaccharide preparations.

To the best of our knowledge, this is the first report describing the *L. saccharina* fucoidan as a powerful *in vivo* inhibitor of tumor-related angiogenesis and tumor growth, as well as heterotypic cell adhesion. These activities appear to correlate with the presence of high levels of O-sulfated fucose units in the fraction **L.s.-1.25**. Lower activity of the fraction **L.s.-1.0** in a number of assays performed could be linked to the presence of mannose and glucuronic acid units in its structure. In light of our findings, the *L. saccharina* fucoidan fraction **L.s.-1.25** may be amenable to be tested in pilot clinical trials.

In order to decipher the structure of the corresponding pharmacophores, a systematic synthesis of the oligosaccharide fragments of different fucoidans [37]–[39] will be required including that of per-O-sulfated oligosaccharides [40], [41]. Assessment of the structure-function relationship of different purified fucoidan fractions may contribute to an improved understanding of the mechanisms underlying the anti-inflammatory, anti-tumor and anti-angiogenic activities of sulfated polysaccharides *in vivo* in order to accelerate their use in clinical settings.
